# Transcription factor c-Myb promotes the invasion of hepatocellular carcinoma cells via increasing osteopontin expression

**DOI:** 10.1186/1756-9966-29-172

**Published:** 2010-12-30

**Authors:** Rong-Xin Chen, Yun-Hong Xia, Tong-Chun Xue, Sheng-Long Ye

**Affiliations:** 1 Liver Cancer Institute and Zhongshan Hospital, Fudan University, Shanghai, China; 2 Key Laboratory of Carcinogenesis and Cancer Invasion (Fudan University), the Chinese Ministry of Education, Shanghai, 200032, China

## Abstract

**Background:**

Specific gene expression is tightly regulated by various transcription factors. Osteopontin (OPN) is a phosphoprotein that mediates hepatocellular carcinoma (HCC) progression and metastasis. However, the mechanism of OPN up-regulation in HCC metastasis remains to be clarified.

**Methods:**

Oligonucleotide array-based transcription factor assays were applied to compare different activities of transcription factors in two human HCC cell lines with different OPN expression levels. The effects of one selected transcription factor on OPN expression were further evaluated.

**Results:**

Eleven transcription factors were over-expressed in metastatic HCC cell line HCCLM6 cells whereas twelve transcription factors were down-regulated. Electrophoretic mobility shift assays (EMSA) and reporter gene assays showed that one of up-regulated transcription factors c-Myb could bind the OPN promoter and increase its transcription activity. In addition, small interfering RNA targeting c-Myb could inhibit OPN expression and significantly decrease migration and invasion of HCCLM6 cells *in vitro*.

**Conclusion:**

Our data first demonstrate that c-Myb has a functionally important role in the regulation of OPN expression in HCC cells, suggesting that c-Myb might be a new target to control HCC metastasis.

## 1. Introduction

Hepatocellular carcinoma (HCC) is one of the most common and aggressive malignancies [1]. Despite of improvements in surgical techniques and perioperative managements, HCC prognosis remains poor due to a 5-year recurrence rate of 50%-70% after resection [2,3]. Thus, it is critical to identify the molecules controlling the invasive and metastatic potential of HCC, which would provide new targets for intervention.

Osteopontin (OPN) is a secreted extracellular matrix protein, which has been linked to tumor progression and metastasis in a variety of cancers including HCC [4,5]. OPN has been identified as the lead gene over-expressed in the metastatic HCC [6]. Increased OPN expression is associated with clinical stage, portending a poor prognosis [7-9]. OPN increases cell proliferation, migration and extracellular matrix invasion *in vitro *through binding its receptors of integrins or CD44 variant. Although OPN has been studied in a number of tumors, the molecular mechanisms of OPN up-regulation in the processes of HCC metastasis are still elusive.

While tumor progression and metastasis are closely related to signaling cascades that transduce and integrate regulatory cues, transcription factors are endpoints of signaling pathways to determine transcription and the extent to which genes are expressed [10]. In addition, some transcription factors including AP-1 [11], SP-1 [12] and Runx [13] have been functionally associated with tumor cell proliferation, growth, differentiation and metastasis in leukemia and solid tumors.

To investigate the possibility that transcription factors regulate OPN expression in HCC metastasis, we applied transcription factor microarrays to compare different activities of transcription factors in two human HCC cell lines with different OPN expression levels. Our data demonstrate that one of up-regulated transcription factors c-Myb plays an important role in the regulation of OPN expression and invasion of HCC cells *in vitro*, suggesting that c-Myb may be a potential target to control HCC metastasis.

## 2. Materials and methods

### 2.1 Cell culture

Human embryonic liver cell line L02 and HCC cell line SMMC-7721 were obtained from Shanghai Institute of Cell and Biology, Chinese Academy of Science and maintained in RPMI supplemented with 10% fetal bovine serum at 37°C with 5% CO_2_. Human metastatic HCC cell line MHCC97-L and HCCLM6 were established at Liver Cancer Institute, Zhongshan Hospital, Fudan University, Shanghai, P.R. China [14] and cultured in DMEM (Invitrogen, Carlsbad, CA) containing 10% fetal bovine serum at 37°C with 5% CO_2._

### 2.2 RNA isolation and reverse transcription-PCR

Total RNA was extracted from cells using TRIzol reagent (Invitrogen, Carlsbad, California) and reverse transcribed into single-stranded cDNA. PCR was done on cDNA using oligo(dT) priming and amplified with the primer pairs for a 436-bp fragment of OPN(forward primer 5'-GGACTCCATTGACTCGAACG-3' and reverse primer 5'-TAATCTGGACTGCTTGTGGC-3') and a 366-bp fragment of Glyceraldehyde-3- phosphate dehydrogenase (GAPDH) (forward primer 5'-ATCCCATCACCATCT TCCAG-3' and reverse primer 5'-GAGTCCTTCCACGA TACC AA-3'). GAPDH was used as a control. Ten microliters of PCR product was analyzed on 2% agarose gels.

### 2.3 RNA isolation and real-time quantitative RT-PCR

RNA was isolated from cells using the TRIzol and was reverse transcribed into cDNA by oligo(dT) primer. QuantiTect SYBR Green PCR kit (Qiagen, Valencia, CA) and DNA Engine Opticon System (MJ Research, Reno, NV) were used for real-time PCR. Data were analyzed with Opticon Monitor software version 1.02. The thermal cycling conditions comprised an initial denaturation step at 95°C for 15 minutes and 45 cycles at 94°C for 15 seconds and 55°C or 57°C for 1 minute. The primers for c-Myb, OPN and GAPDH were shown in Table 1. GAPDH was used as a control and relative expression of genes was determined by normalizing to GAPDH according to the manufacturer's instructions.

**Table 1:** Primers of c-Myb and OPN for real-time quantitative RT-PCR

Gene	Primer sequence (5'→3')	Annealing temperature(°C)	Product length (bp)
c-Myb	TACAATGCGTCGGAAGGTCG	55	201
	GCGGAGCCTGAGCAAAACC		
OPN	GTGGGAAGGACAGTTATGAAACG	57	134
	CTGACTATCAATCACATCGGAAT		
GADPH	ATGACCCCTTCATTGACC	55	131
	GAAGATGGTGATGGGATTTC		

### 2.4 Nuclear extracts and biotin-streptavidin DNA pull-down assay

Oligonucleotide containing biotin on the 5'-nucleotide of the sense strand was used in the PCR amplification for human OPN promoter. The sequences of the primer were as follows: sense strand: 5'biotin-TGGAATACATCCAATTTAAGGGAG-3'; antisense strand 5'-GAATGCACAA CCCAGTAGCAAA-3'; which corresponds to positions -1488 to +185 of the human OPN promoter. Nuclear proteins were isolated from HCC cell line SMMC-7721 and HCCLM6 cells respectively according to manufacturer's directions (NE-PER nuclear and cytoplasmic extraction reagents, Pierce). Protein concentration of the nuclear extract was determined using a BCA assay kit. The nuclear protein was incubated for 1 hour at 25°C with biotinylated PCR product bound to streptavidin agarose beads in protein binding buffer (12% (v/v) glycerol, 24 mM HEPEs PH 7.9, 8 mM Tris PH 7.9, 300 mM KCl, 2 mM EDTANa_2 _0.25 mg/ml poly(dI-dC)). The magnetic beads were washed three times with protein binding buffer and the fractions were eluted with elution buffer (2.0 M NaCl, 20 mM Tris-HCL, pH 8.0, 10%(v/v) glycerol, 0.01%(v/v)Triton X-100, 1.0 mM EDTA, 1 mM dithiothreitol) and were stored at -80°C.

### 2.5 Transcription factor profiling

TranSignal Protein/DNA Microarray I (SuperArray, Bethesda, MD) was used to characterize the transcription factor profiles of SMMC-7721 and HCCLM6 cells. The chip included 254 transcription factors. The nuclear protein from DNA pull-down assay was incubated for 30 minute at 15°C with the TranSignal probes, and then the compounds was washed three times with wash buffer and eluted with elution buffer to get the probes. When used, probes from three independent expreriments were taken and mixed by equal volume. Then, probes were hybridized with microarrays performed according to the manufacturer's instructions as described previously [15].

### 2.6 Electrophoretic Mobility Shift Assays (EMSA)

Nuclear extract preparation and electrophoretic mobility shift assays were conducted as described previously [12]. The oligonucleotides containing c-Myb-binding site were used in EMSA according to the manufacturer's instructions (Chemiluminescent nucleic acid detection module, Pierce). The oligonucleotides were labeled with biotin according to standard protocols. The sequences of the oligonucleotides were as follows: 5'Biotin-TAC AGGCATAACGGTTCCGTAGTGA-3'. The point mutant (underlined) of oligonucleotides was constructed: 5'Biotin-TACAGGCATATCGGTTCCGTAGTGA-3'. The oligonucleotides was annealed to its complementary oligonucleotides and incubated with nuclear proteins for 30 minute at 25°C. Samples were run on a 6% polyacrylamide gel, which was transfered into Nylon member and then blocked and washed. Bands were detected by chemiluminescent method.

### 2.7 Luciferase Assay

The OPN promoter was amplified by from HCCLM6 cells as described above [12]. The amplified OPN promoter encompassed all c-Myb binding sites to test transcriptional activity [16]. The resulting 1673-bp fragment (-1488 to +185) was ligated into the Kpn I and Xhol I sites of the pGL3-Basic luciferase reporter vector (Promega, Madison, WI). In brief, 4 x10^5 ^cells were seeded the day before transfection. Then, 2 ug of plasmid DNA and 4 ul of LipofectAMINE 2000 (Invitrogen, Carlsbad, CA), diluted with Opti-MEM, were mixed gently and incubated with cells. Together, the small RNA interference (siRNA) targeting c-Myb was chemically synthesized and tranfected into cells using LipofectAMINE 2000. Culture medium was changed after 6 hours of transfection. Cells were washed with PBS and lysed in lysis buffer after 36 hours after transfection according to the manufacturer's instructions. Luciferase activity was measured by luminometer (Lumat LB970). Luciferase activity was normalized for β-Galactosidase (pSV-β-Galactosidase Control Vector). Experiments were performed in triplicate.

### 2.8 Small Interfering RNA (siRNA)

The Sequence targeted to the site of c-Myb mRNA (GeneBank Accession No. NM_005375) were designed without off-target effects. The sense and antisense strands of c-Myb siRNAs were 5'-GGACGAACUGAUAAUGCUATT-3' and 5'-UAGCAUUAU CAGUUCGUCCAG-3', respectively. For transfection of the HCC cells, c-Myb siRNA or a negative-control mismatch sequence (scramble siRNA) was transfected with LipofectAmine 2000 (Invitrogen, Carlsbad, CA) according to the manufacturer's instructions.

### 2.9 Western blot

Total protein extraction from cultured cells was used in electrophoresis and western blot. Briefly, twenty micrograms of total protein were separated by standard SDS-PAGE and then transferred to PVDF membranes. The membranes were washed, blocked, and incubated with the specific primary antihuman antibodies against OPN (1:800) or against c-Myb (1:500), anti-GADPH antibody (1:5000) (Santa Cruz), followed by incubation with horseradish peroxidase-conjugated secondary antibodies. The reactions were detected by enhanced chemiluminescence assay.

### 2.10 Matrigel invasion assay and migration assay

The invasive ability of the transfected cells was determined by the Matrigel (BD Pharmingen) coated 24-well transwell chambers with upper and lower culture compartments separated by polycarbonate membranes with 8-um pore(Costar, NY, USA). The bottom chamber was filled with DMEM containing 10% FBS as a chemoattractant. The transfected cells (1 × 10^5^) were seeded on the top chamber and incubated at 37°C with 5% CO_2_. After 40 hours, the cells removed from the upper surface of the Matrigel by scrubbing with a cotton swab and cells that migrated to the underside of the membrane were stained with Giemsa (Sigma). Five high-power fields were counted and the mean number of cells per field was calculated. The migration assay was similar to the invasion assay only without Matrigel and lasted for 18 hours. The experiments were performed in triplicate.

### 2.11 Statistical analysis

Statistical analyses were performed by the Statistical Package for the Social Sciences version 11.5 (SPSS, Inc., Chicago, IL). Data were expressed as means ± SD, and analyzed using the two-tailed Student's t-test or the Analysis of Variance (ANOVA). The level of significance was set at *P *< 0.05.

## 3. Results

### 3.1 Differential activity of transcription factors in two HCC cell lines with different OPN expression levels

**Figure 1:**
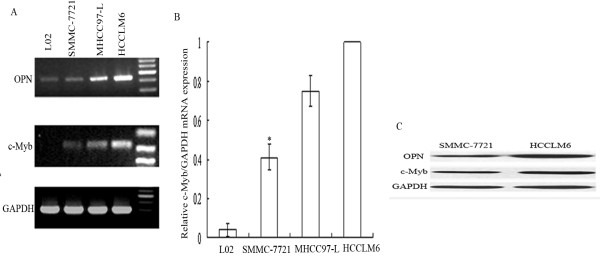
**Verification of difference of OPN and c-Myb expression in HCC cell lines**. HCCLM6 cells expressed high level of OPN and c-Myb compared with SMMC-7721 cells. (A) Relative OPN and c-Myb mRNA levels in different cell lines by RT-PCR analysis. (B) Real-time quantitative PCR analysis confirmed the difference of c-Myb mRNA expression in different cell lines. Graph depicted relative expression of OPN mRNA normalized to that of GAPDH. The mRNA expression of c-Myb in HCCLM6 was used as control. Data expressed as means ± SD (*P < 0.05, SMMC-7721 *vs*. HCCLM6). (C)Western blot analysis of OPN and c-Myb protein expression in HCC cell line SMMC-7721 and HCCLM6. Blot was representative of three experiments.

Compared to the weakly tumorigenic and non-metastastic HCC cell line SMMC-7721 cells, HCCLM6 cells with highly metastatic potential expressed high level of OPN (Figure 1A, C). With > 2-fold or < 0.5-fold expression as the cutoff point, analysis of transcription factor profiles revealed that eleven transcription factors including c-Myb, MAZ and E4BP4 were highly up-regulated meanwhile twelve transcription factors were reduced in HCCLM6 cells (Table 2). In particular, the expression of c-Myb was at a high level in metastatic HCC cell line HCCLM6 and MHCC97-L cells, and at a much lower level in SMMC-7721 cells, and barely detectable in normal cell line L02 cells. Corresponding to different OPN expression level (HCCLM6 > MHCC-97-L> SMMC-7721), the expression level of c-Myb increased sharply in HCCLM6 cells (Figure 1A). Similar results were obtained in real-time PCR analysis. When normalized to the internal standard control, mRNA expression of c-Myb in HCCLM6 cells was significantly higher than SMMC-7721 cells (Figure 1B). Similar to the result of mRNA expression, the difference of c-Myb protein expression between HCCLM6 and SMMC-7721 cells was also significant. (Figure 1C)

**Table 2:** Differential activity of transcription factorsin two HCC cell lines (SMMC-7721, HCCLM6) with different OPN expression levels (> 2 fold or <0.5-fold change)

Name	HCCLM6/SMMC-7721 ratio	Description
Up-regulation		
MAZ	3.10	MYC-associated zinc finger protein
E4BP4	2.86	nuclear factor, IL- 3 regulated
c-Myb	2.80	v-myb myeloblastosis viral oncogene
GATA-2	2.74	GATA binding protein 2
TEF1	2.73	activator
PEBP2	2.39	polyoma enhancer binding protein 2
Smad3/4	2.27	MADH3/4
IRF-1/2	2.21	interferon regulatory factor 1/2
PEBP	2.13	polyoma enhancer binding protein
GAG	2.13	amyloid precursor protin (APP) regulator
ADR1	2.10	alcohol dehydrogenase regulatory gene 1
Down-regulation		
NF-E2	0.19	nuclear factor (erythroid-derived 2), 45 kDa
EGR	0.21	early growth response
C/EBPα	0.22	CCAAT/enhancer binding protein alpha
E2F-1	0.28	E2F transcription factor 1
CYP1A1	0.30	cytochrome P450-c
HiNF-A	0.31	A nuclear protein
Sp1	0.31	Sp1 transcription factor
E12/E47	0.31	enhancer binding factors E12/E47
PARP	0.34	poly(ADP-ribose) synthetase/polymerase
ELK1	0.34	member of ETS oncogene family
E4F1	0.34	E4F transcription factor 1

### 3.2 Transcription factor c-Myb contributing to transcription activation of the OPN promoter in HCCLM6 cells

**Figure 2:**
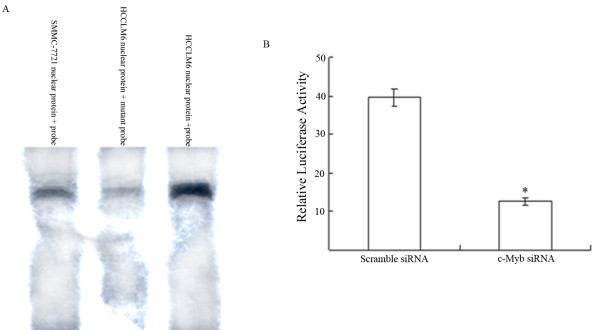
**Electrophoretic mobility shift sssays (EMSA) of c-Myb binding to OPN promoter and transient transfection analysis of OPN promoter activity**. (A). EMSA were performed using nuclear extract prepared from SMMC-7721 and HCCLM6 cells. Assays utilized a labeled probe of 25-nt fragment containing the area of c-Myb binding site in the OPN promoter or a mutant form of the c-Myb binding site (c-Myb-binding site TAACGG was mutated to TATCGG). The blot was representative of three experiments. (B) To confirm the role of c-Myb in the increased OPN protein expression in HCCLM6 cells, Human OPN promoter (-1488 to +185 nt) was cloned into the pGL3-basic luciferase reporter vector. The OPN promoter reporter constructs were transfected into HCCLM6 cells. In certain instances, c-Myb siRNA or scramble siRNA was co-transfected. Luciferase activity was normalized to that of β-galactosidase activity. Data are presented as means ± SD of three experiments. (*P < 0.05, c-Mb siRNA-treated group *vs*. scramble siRNA group).

Having shown that c-Myb was over-expressed in HCCLM6 cells, we next sought to establish whether it has a functionally important role in the regulation of OPN expression. To establish if functional c-Myb is present in HCCLM6 cells, nuclear extracts were incubated with the oligonucleotides containing c-Myb-binding site and the formation of specific complexes was determined by EMSA. A double-stranded biotin-labeled oligonucleotides encompassing the c-Myb site or a mutant form of the c-Myb site in the OPN promoter were used. When nuclear extracts from HCCLM6 cells was incubated with the oligonucleotides containing c-Myb site, a specific retarded complex was observed. In contrast, incubation with the oligonucleotides containing mutant c-Myb site significantly abrogated binding (Figure 2A). In addition, the oligonucleotides containing the c-Myb site incubated with nuclear extracts from SMMC-7721 cells formed a weakly specific retarded complex (Figure 2A). These data demonstrate that the c-Myb site in the OPN promoter can be specifically bound by transcription factor c-Myb in HCCLM6 cells.

To further determine whether the c-Myb site in the OPN promoter was required for transcription activation, HCCLM6 cells were transfected with an OPN promoter reporter plasmid. To assess whether down-regulation of c-Myb could suppress the transcription activity of the OPN promoter, HCCLM6 cells were co-transfected with the OPN promoter reporter and siRNA targeting c-Myb. Inhibition of c-Myb expression by siRNA significantly decreased OPN promoter activity in HCCLM6 cells. In contrast, co-transfection of the OPN promoter reporter and a scramble siRNA had no effect on the activity of the OPN promoter (Figure 2B). These data demonstrate that c-Myb is essential for transcription activity of OPN in HCCLM6 cells.

### 3.3 OPN expression was down-regulated after c-Myb was inhibited in HCCLM6 cells

**Figure 3:**
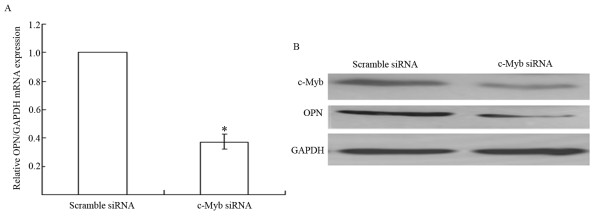
**The effect of c-Myb on OPN expression of HCCLM6 cells**. (A) OPN mRNA expression in HCCLM6 cells transfected with c-Myb siRNA was significantly decreased. (*P < 0.05, *vs *control). The mRNA expression of OPN in cells transfect with scramble siRNA was used as control. (B) OPN protein expression in HCCLM6 cells transfected with c-Myb siRNA was significantly reduced compared with cells transfected with sramble siRNA. Blot was representative of three experiments.

To further validate c-Myb regulating OPN expression in HCCLM6 cells, we examined the level of OPN expression in HCCLM6 cells transfected with siRNA targeting c-Myb. The results showed that inhibition of c-Myb expression by siRNA significantly decreased OPN mRNA and protein expression (Figure 3A, B), suggesting that c-Myb contributes to the regulation of OPN expression in HCCLM6 cells.

### 3.4 Migration and invasion of HCCLM6 cells in vitro were inhibited by c-Myb siRNA

**Figure 4:**
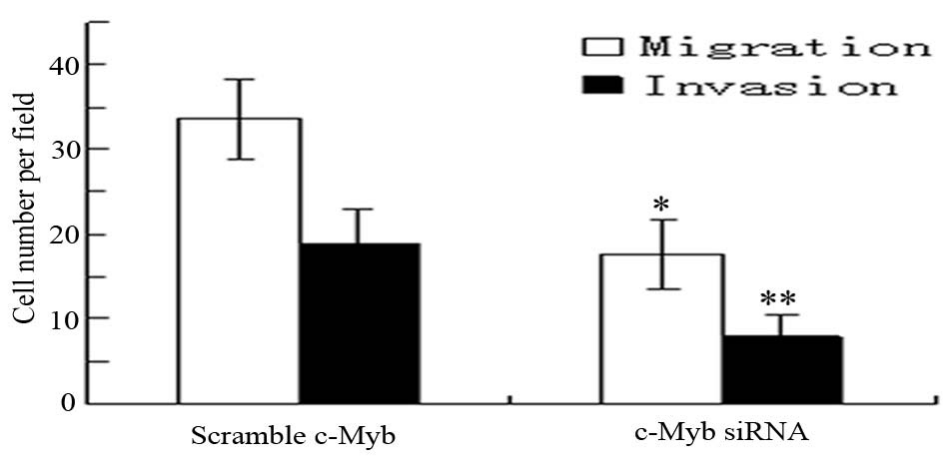
**Migration and invasion of HCCLM6 cells in response to transfection of c-Myb siRNA**. The c-Myb siRNA could significantly inhibit the migration and invasion of HCCLM6 cells compared with cells treated with scramble siRNA (*P < 0.05). The migration and invasion assays were assessed by transwell chambers. Data were expressed as means ± SD of three experiments.

As migratory and invasive behaviors are the indicators of the metastatic potential, we examined migration and invasion of HCCLM6 cells *in vitro *using the transwell assay after c-Myb expression was inhibited by c-Myb siRNA. The average numbers of HCCLM6 cells transfected with c-Myb siRNA migrating toward the conditioned medium or invading through the Matrigel were significantly fewer than those transfected with scramble siRNA (Migration assay: 17.60 ± 4.04 *vs *33.60 ± 4.67, *P *< 0.05; Invasion assay: 8.00 ± 2.55 *vs *18.8 ± 4.15, *P *< 0.05, Figure 4), This result showed that the capability of migration and invasion in HCCLM6 cells was significantly decreased after inhibition of c-Myb, suggesting that c-Myb is an important contributor to the migration and invasion of HCC cells.

## Discussion

Metastasis remains one of the major challenges for HCC patients undergoing various therapies including liver resection, local ablation and chemoembolization [2,3]. Previous work at our institute has shown that OPN gene is over-expressed in the metastatic HCC [6]. In this study, we searched for transcription factors that were correlated with OPN expression in HCC cells and revealed that transcription factor c-Myb was positively associated with OPN expression in HCC cells, which can bind the OPN promoter and increase its transcription activity. Inhibition of c-Myb by siRNA decreased the transcription activity of the OPN promoter, reduced the expression of OPN, and compromised the ability for migration and invasion of HCC cells. Therefore, our results demonstrate that c-Myb plays an important role in regulating OPN expression in HCC cells, suggesting c-Myb might be a novel target for therapeutic intervention.

OPN is known to mediate correlates of metastatic biology in a variety of cancers including HCC. Thus, modulating OPN expression might be a novel approach of suppressing tumor metastasis [17-19]. Transcription factors are located at endpoints of signaling pathways and integrate various stimuli to determine which genes are expressed or suppressed [10]. To search for the determinant transcription factors regulating OPN in HCC, we used transcription factor microassays to compare differential activities of transcription factors between two HCC lines with different OPN expression level. Through microarray analysis, we found that eleven transcription factors were highly expressed meanwhile twelve were down-regulated in metastatic HCC cells. Transcription factor c-Myb was selected for further investigation. The reasons are the following: (1) after predicting the potential transcription factors in the OPN promoter in the TRANSFAC database http://www.gene-regulation.com and searching the reported transcription factor which can bind to the OPN promoter in the literature [20], we have found that among the eleven up-regulated transcription factors, c-Myb and IRF-1 have the definitive binding sites in the OPN promoter. Although the rests of transcription factors were up-regulated in gene-chip analysis, they lacked the reported binding site in the OPN promoter and may act by the way of combining with co-activators or other transcription factors, and then together binding to specific sites of the OPN promoter. (2) Interestingly, Schultz J and colleagues [21] have reported that differential capability of c-Myb binding to -443T/C osteopontin promoter influences osteopontin gene expression in melanoma cells, suggesting the importance of c-Myb regulating OPN expression in tumor progression. In this study, c-Myb expression increased corresponding to OPN levels in different HCC cell lines, suggesting that c-Myb is associated with OPN expression. The differences of OPN expression might reflect the differential activities of c-Myb among HCC cell lines. EMAS and luciferase assays further demonstrated that c-Myb is essential for transcription activity of OPN in HCC cells.

The transcription factor c-Myb has a key role in regulating the exquisite balance among cell division, differentiation and survival and has now been identified as an oncogene involved in some human leukemia and solid cancers [22-24]. Recently, it is reported that oncogene c-Myb participates in the process of hepatitis B virus-induced liver carcinogenesis [21]. When inappropriately expressed, c-Myb appears to activate important gene targets to promote cancer progression and metastasis. These genes include cyclooxygenase-2 (COX-2) [25], Bcl-2, BclX(L) [26] and c-Myc [27], which influence diverse processes such as angiogenesis, proliferation and apoptosis. As for HCC, Yang et al [28] has documented that increased expression of c-Myb and Sp1 binding to the methionine adenosyltransferase 2A (MAT2A) promoter contribute to the up-regulation of MAT2A expression. MAT2A can catalyze the formation of S-adenosylmethionine to facilitate HCC growth. In the present study, we first demonstrate that c-Myb is a new transcription factor of regulating OPN expression in HCC cells, providing at least one mechanism for up-regulation of OPN expression in HCC invasion and metastasis.

Considering transcription factors including AP-1, Sp-1, v-Src, Runx and Tcf-4 participating in the transcription regulation of OPN in other types of cancers [20,29], and transcription factor along with co-activators or co-repressors strategically binding to specific sites of target gene promoters [30], it is possible that c-Myb interacts with other transcription factors to modulate the OPN expression in HCC cells. This requires further validation.

Apart from demonstrating the function of c-Myb in the regulating OPN expression in HCC cells, we also showed that down-regulation of c-Myb by siRNA decreased OPN expression and also inhibited the migration and invasion of HCCLM6 cell *in vitro*, indicating that modulating OPN expression by targeting c-Myb might be a new approach for intervening HCC invasion and metastasis. Antisense oligodeoxynucleotides targeting c-Myb, a dominant negative c-Myb or c-Myb vaccine has shown an effective approach for therapy of c-Myb dependent haematopoietic and epithelial malignancies [31-33].

In summary, our data demonstrate that transcription factor c-Myb is over-expressed in the metastatic HCC cells and has a functionally important role in the regulation of OPN expression, suggesting that c-Myb might be a new target for therapeutic intervention in the HCC invasion and metastasis by modulating OPN expression.

## Competing interests

The authors declare that they have no competing interests.

## Authors' contributions

CRX and SLY designed the study. CRX, YHX and TCX performed experiments. CRX drafted the manuscript. All authors read and approved the final manuscript.
